# Does 3D-speckle tracking echocardiography improve prediction of major cardiovascular events in a multi-ethnic general population? A Southall and Brent Revisited (SABRE) cohort study

**DOI:** 10.1371/journal.pone.0287173

**Published:** 2023-06-27

**Authors:** Lamia Al Saikhan, Chloe Park, Therese Tillin, Siana Jones, Jamil Mayet, Nish Chaturvedi, Alun Hughes

**Affiliations:** 1 Department of Cardiac Technology, College of Applied Medial Sciences, Imam Abdulrahman Bin Faisal University, Dammam, Saudi Arabia; 2 Department of Population Science & Experimental Medicine, UCL Institute of Cardiovascular Science, MRC Unit for Lifelong Health and Ageing, University College London, London, United Kingdom; 3 NIHR Imperial Biomedical Research Centre, Imperial College London and Imperial College Healthcare NHS Trust, Hammersmith Hospital, London, United Kingdom; Oswaldo Cruz Foundation, BRAZIL

## Abstract

3D-speckle tracking echocardiography(3D-STE) allows simultaneous assessment of ejection fraction(EF) and multidirectional strains, but its prognostic utility in the general population is unknown. We investigated if 3D-STE strains predicted a composite of major cardiac endpoints(MACE) beyond cardiovascular risk factors(CVDRF), and whether they were superior to 3D-EF. 529 participants in SABRE, a UK-based tri-ethnic general population cohort (69±6y; 76.6% male) with acceptable 3D-STE imaging were studied. Associations between 3D-EF or multidirectional myocardial strains and MACE(coronary heart disease(fatal/non-fatal), heart failure hospitalization, new-onset arrhythmia and cardiovascular mortality) were determined using Cox regression including adjustment for CVDRF and 2D-EF. Whether 3D-EF, global longitudinal strain(3D-GLS) and principle tangential strain(3D-PTS/3D-strain) improved cardiovascular risk stratification over CVDRF was investigated using a likelihood ratio test on a series of nested Cox proportional hazards models and Harrell’s C statistics. During follow-up(median, 12y), there were 92 events. 3D-EF, 3D-GLS and 3D-PTS and 3D-RS were associated with MACE in unadjusted and models adjusted for CVDRF but not CVDRF+2D-EF. Compared to 3D-EF, both 3D-GLS and 3D-PTS slightly improved the predictive value over CVDRF for MACE, but the improvement was modest(C statistic increased from 0.698(0.647, 0.749) to 0.715(0.663, 0.766) comparing CVDRF with CVDRF +3D-GLS). 3D-STE-derived LV myocardial strains predicted MACE in a multi-ethnic general population sample of elderly individuals from the UK; however the added prognostic value of 3D-STE myocardial strains was small.

## Introduction

Assessment of global left ventricular (LV) systolic function is key to evaluating prognosis and in determining therapeutic strategies in cardiovascular disease (CVD) [1]. Reduced ejection fraction (EF) is associated with unfavourable outcomes in populations with established CVD [2], and in the general population [3]. However, this parameter suffers from inherent limitations,[1] and may have poorer predictive value in people with less severe CVD [4].

LV strain by 2-dimensional (2D) speckle-tracking echocardiography (STE), particularly global longitudinal strain (GLS), may be superior to EF for prognosis in patients with a range of cardiac disease, [5] and its use, when available, has been advocated in some recent guidelines [6,7]. Nevertheless, myocardial deformation is an intrinsically 3-dimensional (3D) process and, in theory, 3D-STE may have advantages over 2D-STE as it allows simultaneous assessment of LV myocardial deformation/mechanics in three orthogonal directions, namely longitudinal (LS), circumferential (CS) and radial (RS) strains. It also enables calculation of principle tangential strain (3D-PTS), which characterises LV shortening along the main direction of contraction [8]. A study in a sample of inpatients referred for echocardiography reported that 3D-GLS achieved better prediction of cardiovascular mortality than 3D-EF, [9] however, the diagnostic and prognostic usefulness of 3D-PTS remains to be established, particularly in general population samples, and concerns exist over its dependence on image quality, and its low temporal and spatial resolution [8,10,11].

Accordingly, we investigated whether multidirectional strain components assessed by 3D-STE predicted major adverse cardiac endpoints (MACE) in a multi-ethnic UK community-based sample. We also evaluated whether 3D-strains were superior to 3D-EF and the additional prognostic utility of 3D-STE over and above established CVDRF and 2D-EF.

## Materials and methods

### Study population

**Fig 1:**
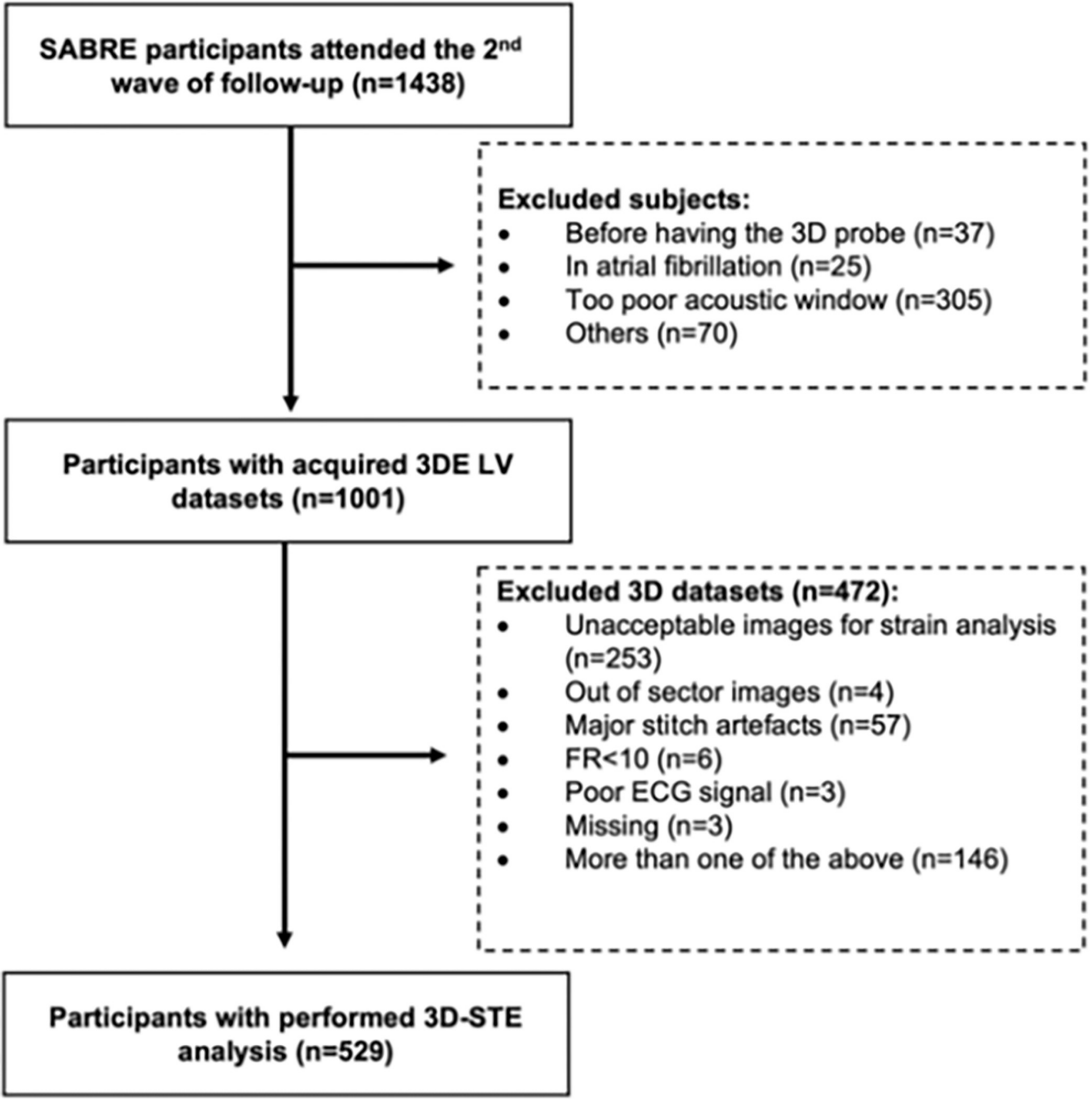
Flow diagram of the study population. Abbreviations: FR, frame rate; 3DE, 3D echocardiography; 3D-STE, 3D speckle-tracking echocardiography; LV, left ventricular.

The design of Southall and Brent revisited (SABRE) study has been described previously [12,13]. Briefly, all traceable surviving participants from the baseline studies (1988–1991) were invited to attend the 20-year follow-up clinical examination (2008–2011) at St Mary’s Hospital, London. 1438 participants (average age 69.6y) attended the clinic following an overnight fast. All participants underwent comprehensive clinical investigations and completed health and lifestyle questionnaires [13]. Of these 1001 people underwent 3D echocardiography (3DE); people with atrial fibrillation (AF), inadequate acoustic windows or other reasons precluding echocardiography were excluded as shown in **Fig 1**. 3D-STE LV analysis was acceptable in 529 participants (i.e. only 53% of the 1001 participants undergoing 3DE); more details on the feasibility of 3D-STE in SABRE have been reported elsewhere [13,14].

The study was approved by St Mary’s Hospital Research Ethics Committee (07/H0712/109) and was performed in accordance with the principles of the Helsinki Declaration. Written informed consent was obtained, including consent for primary care medical record review and data linkage.

### Echocardiographic imaging and analysis

Echocardiography was performed by two experienced sonographers using a commercially available system (Phillips iE33 equipped with a S5-1 phased array and a X3-1 matrix array transducers). Full detail of SABRE standardized echocardiography imaging, quality control and analysis protocol has been described previously [13].

#### Conventional echocardiography

Chamber dimensions and wall thickness from 2D-guided M-mode were measured from parasternal long-axis view from which LV mass was calculated following ASE recommendations [7]. LV volumes were calculated by the Teichholz formula. This method is now considered sub-optimal, but when the study was designed it was consistent with contemporary guidelines and its use maintained compatibility with methods used for previous visits. Tissue-Doppler analysis was performed for lateral and septal mitral annulus motion and peak longitudinal systolic velocity (s′), and peak early and late mitral annular relaxation velocities (e′, a′) were calculated and averaged. Mitral inflow early and its deceleration time, and late diastolic velocities were measured by PW Doppler with a sample volume placed at the tip of mitral valve leaflets and E/A ratio was calculated [15]. E/e′ was calculated as an index of LV filling pressure [15]. Presence of any valve disease was ascertained.

#### 3D speckle tracking echocardiography

Image settings were optimized and 3D LV full-volume dataset of 4 sub-volumes (wide-angled 93°×80°) was obtained from the apical position acquired over 4 cardiac cycles. The presence of an acceptable ECG signal was checked before accepting the loop. Scans were stored on a secure fileserver in DICOM or native format.

**Fig 2:**
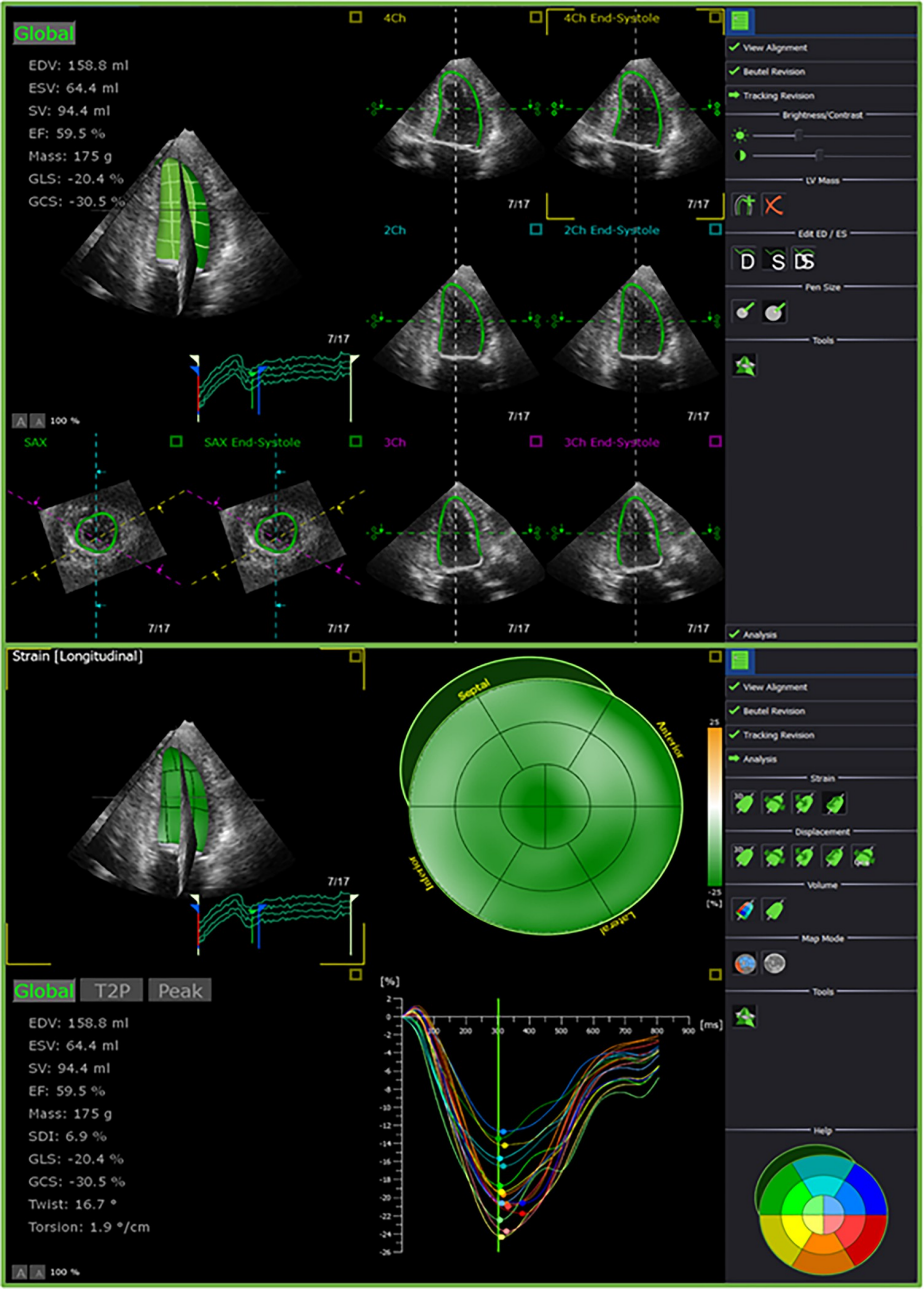
An example of 3D speckle-tracking echocardiography (3D-STE) myocardial deformation analysis performed using TomTec 4D LV-Analysis.

3D-STE LV myocardial deformation analysis was performed offline using TomTec 4D LV-Analysis (TomTec Imaging Systems, Munich, Germany) according to a pre-specified protocol, (**Fig 2**) described in detail elsewhere [13]. In brief, the quality of 3D LV datasets was assessed and defined as good, fair, adequate, poor or unacceptable. The software automatically selected and displayed three standard apical views and one short-axis view. Alignment of the longitudinal axis of the LV in all apical views were further modified manually if needed using two anatomical landmarks (i.e. the mitral valve annulus and the apex). The software automatically tracked the LV endocardial borders at end-diastole and manual adjustments were performed when needed, although user interaction was kept to a minimum to minimize variability in measurements. The software then tracked the endocardium throughout the cardiac cycle in 3D space from which the 3D LV endocardial shell was constructed. The tracking quality was visually evaluated as the software did not provide automatic evaluation for image tracking adequacy. When the tracking of LV endocardial boundaries appeared suboptimal, it was further adjusted manually. The software then divided the LV into 16 segments and provided curves as well as maps of global and segmental volumetric and deformation indices.

Obtained LV deformation indices were 3D-GLS, global circumferential strain (3D-GCS), and peak averaged segmental LS, CS, RS and 3D-PTS, twist and torsion. 3D-PTS represents the major direction and magnitude of deformation in which no shear strain occurs; it provides an integrated measure of LV 3D deformation that is related to the geometry and active stress generation by cardiac myofibres [16]. Global strain measures were computed based on the entire contour length of longitudes (i.e. averaged over the myocardium), whereas the peak averaged segmental strain measures were the calculated averages from the individual 16-segment values. 3D-EF and volumes were also measured. The key exposures in terms of LV functional indices were: 3D-EF, 3D-GLS, 3D-GCS, 3D-PTS, 3D-RS, twist and torsion. A more negative strain (i.e. shortening) is indicative of better systolic function, so to facilitate comparisons of hazard ratios (HR), 3D-GLS, 3D-GCS and 3D-PTS were multiplied by -1 before calculating HR. Details of the reproducibility of these parameters in SABRE have been reported previously [13].

#### Follow-up and outcome

Participants were followed up until April 2021 or the time of the event for morbidity and mortality by the Hospital Episode Statistics (HES) and UK Office of National Statistics (ONS). Causes of deaths were defined according to the International Classification of Disease (ICD) diagnostic codes (ICD-9 or ICD-10). HES provided information regarding admissions, discharge, and the ICD diagnostic codes and operation codes (i.e. the Office of Population Censuses and Surveys (OPCS) classification of interventions and procedures).

The primary outcome was a MACE comprising new onset fatal and non-fatal coronary heart disease (CHD); this was defined as angina, MI or its complications and atherosclerotic heart disease (diagnostic codes, ICD-9: 410–415, ICD-10: I200–I259), or emergent coronary revascularization interventions (OPCS codes: K401-K469, K491-K504, K751-K759)), HF hospitalization (ICD-9: 428, ICD-10: I50), new-onset AF (ICD-9: 4273, ICD-10: I48), ventricular fibrillation (ICD-9: 4274, ICD-10: I490), and cardiovascular mortality (ICD-9 codes 390–460, 410–415 and 430–439, or ICD-10 codes I100-I990, I200-I259 and I600-I699). CHD events that occurred before SABRE clinic attendance were identified from self-report and primary-care medical record review. Medical record review was undertaken by trained research team members in local general practices and at primary care trusts. CHD events identified from primary care record review were adjudicated by two senior physicians according to the ASCOT study criteria [17].

#### Statistical analysis

Continuous variables describing the sample are presented as mean±SD or median (interquartile range) as appropriate. Categorical variables are presented as counts (percentages). Differences between two groups were compared using a two-sample *t*-test or a Mann-Whitney test for continuous variables as appropriate and a χ^2^ test for categorical variables. Analyses were performed using Stata (17.0, StataCorp LLC, USA).

Cumulative hazard curves were created using the Nelson-Aalen method and the median was used to dichotomize continuous predictive variables and statistical testing was performed using a log-rank test. For follow-up time, individuals were censored at the end of the follow-up (April 2021) or at the time of event of interest.

Multivariable associations between 3D-STE-derived LV myocardial deformation/strain indices and outcomes were determined using Cox proportional hazard models, and HR(95% confidence interval (CI)) were reported. Cox regression model diagnostics were performed to check that assumptions of proportional hazards were satisfied. Unadjusted and multivariable-adjusted HR were calculated. Covariates in multivariable Cox regression models were selected *a priori* based on components of the Framingham risk score (FRS) plus cystatin C as a measure of renal function, but variables with no impact on associations were removed from the final models to preserve model stability relative to number of events (model-1: age, sex and ethnicity; model-2: model-1 plus hypertension, diabetes mellitus (DM), body mass index, smoking, cystatin-C, LV-3DE image-quality score; model-3: model-2 plus prior history of CHD); model-4: model-2 plus 2D-EF). Non-cardiovascular death was considered a competing risk, and therefore competing risk analysis using Fine‐Gray estimators was performed and results presented as a sensitivity analysis.

While heart rate (HtR) is not routinely included in most risk scores, since HtR is plausibly associated with some of the exposures and the outcome, a sensitivity analysis was performed by adding HtR to model-3. The possibility that prior history of CHD modified associations was investigated by testing for a statistical interaction and a further sensitivity analysis was performed by excluding individuals with prior history of CHD. Key exposures were standardized to facilitate comparisons between HR.

Harrell’s C statistics obtained from Cox proportional hazards models were used to assess the discrimination of 3D-STE derived LV myocardial strain indices for predicting the outcome to indicate which of the strain components might identify individuals at higher risk. The added incremental value of 3D-EF, 3D-GLS and 3D-PTS in improving cardiovascular risk stratification by FRS was investigated using a likelihood ratio test on a series of nested Cox proportional hazards models for goodness-of-fit (assessed using visual inspection and summarised as χ^2^ values), and AIC and BIC were calculated as measures that combine fit and complexity [18]. Continuous net reclassification improvement (NRI) and integrated discrimination improvement (IDI) were also calculated by adding each of these measures to the clinical predictors from the FRS using the custom-written incrisk package in Stata. FRS was used as a reference as it has been validated in SABRE and performs at least as well as newer risk scores such as QRISK2 [19]. Since FRS would not normally be calculated for the purposes of prognostication on people with existing CHD we also performed a sensitivity analysis by excluding these individuals. For all analyses, a two-tailed *P*-value of <0.05 was considered statistically significant.

## Results

### Population characteristics

The age of the study sample was 69±6y and 405 (76.6%) were male (**Table 1)**. During follow-up (median, 12y, range, 0.5-13y), there were 92 MACE (incidence rate = 0.0163 person-yea). The majority of participants had “normal” LV systolic function (3D-EF>50%). Participants with MACE tended to be older, more likely to be men, South Asian, obese, have hypertension, DM, and a history of CHD. In individuals experiencing MACE, LV systolic function, (determined by 2D-EF or 3D-EF) was more impaired, and cavity volumes and LV mass were larger. 3D-GLS, 3D-PTS and 3D-RS were more impaired in participants with MACE compared to those without. Participants in whom 3D-STE could not be obtained were older, more likely to be South Asian, heavier, and to have hypertension, DM, and history of CHD (**S1 Table in S1 File**).

**Table 1:** Characteristics of the SABRE population (n = 529).

	All (n = 529)	MACE (+) (n = 92)	MACE (-) (n = 437)	*P*
**Demographics**				
Age, years	69.1±6.1	69.9±6.4	69.0±6.0	0.177
Male, n(%)	405(76.6)	83(90.2)	322(73.7)	**0.001**
Ethnicity, n(%)				**0.002**
Europeans	273(51.6)	38(41.3)	235(53.8)	
South Asians	151(28.5)	40(43.5)	111(25.4)	
African Caribbean	105(20.0)	14(15.2)	91(20.8)	
Height, cm	168.7±8.2	169.6±7.5	168.6±8.3	0.270
Body mass index, kg/m^2^	26.1±3.5	26.4±3.4	26.0±3.5	0.356
Waist: hip ratio	0.96±0.1	0.99±0.1	0.96±0.1	**<0.0001**
**Clinical history**				
Systolic blood pressure, mmHg	140.2±17.9	144.3±17.9	139.3±17.7	**0.013**
Diastolic blood pressure, mmHg	76.5±9.6	75.3±9.3	76.8±9.7	0.171
Heart rate, bpm	67.2±11.4	65.5±12.8	67.6±11.1	0.115
Known hypertension, n(%)	301(56.9)	70(76.1)	231(52.9)	**<0.0001**
Known diabetes, n(%)	118(22.3)	30(32.6)	88(20.1)	**0.009**
Prior coronary heart diseases, n(%)	54(10.2)	24(26.1)	30(6.9)	**<0.0001**
Smoking status, n(%)				0.46
Never	285(54.1)	47(51.1)	238(54.7)	
Ex.	201(38.1)	35(38.0)	166(38.2)	
Current	41(7.8)	10(10.9)	31(7.1)	
**Medications**				
Anti-diabetic drugs, n(%)	71(13.4)	19(20.7)	52(11.9)	**0.025**
Lipid lowering drugs, n(%)	247(46.7)	57(62.0)	190(43.5)	**0.001**
**Blood biochemistry**				
Fasting blood triglycerides, mmol/l	1.2±0.6	1.3±0.6	1.2±0.6	0.112
Fasting blood cholesterol: HDL ratio	3.6±1.0	3.6±1.1	3.6±1.0	0.795
HbA1c, %	6.1±0.9	6.3±1.0	6.1±0.8	**0.028**
Cystatin C, mg/l	1.0±0.3	1.1±0.3	1.0±0.3	**0.013**
NT-proBNP, pg/ml	80[46–144]	113[55–218]	75.5[44.5–129]	**0.0003**
Troponin, pg/ml	6.5[4.0–9.9]	8.0[5.8–12.7]	6.1[3.8–9.1]	**0.0002**
**Conventional echocardiography**				
LVIDd, cm	4.5±0.4	4.6±0.5	4.4±0.4	**0.001**
LVIDs, cm	2.9±0.5	3.1±0.6	2.9±0.4	**0.0002**
IVSd, cm	1.1±0.2	1.2±0.2	1.1±0.2	**0.059**
PWd, cm	1.0±0.2	1.1±0.2	1.0±0.2	**0.018**
Relative wall thickness	0.5±0.1	0.5±0.1	0.5±0.1	0.695
2D-EF, %	62.5±9.4	58.8±11.6	63.3±8.7	**0.0008**
2D-EDV, ml/m^2^	49.6±9.9	52.6±11.8	48.9±9.4	**0.008**
2D-ESV, ml/m^2^	18.8±7.3	22.1±10.3	18.1±6.3	**0.0005**
LV mass indexed to BSA, g/m2	92.3±22.4	100.5±26.2	90.6±21.2	**0.001**
LV mass indexed to height^2.7^, g/h^2.7^	41.6±10.8	45.4±13.1	40.8±10.1	**0.002**
E wave, cm/s	62.7±15.6	64.2±17.4	62.4±15.2	0.372
A wave, cm/s	74.8±16.4	79.2±15.3	73.9±16.5	**0.005**
E/A ratio	0.86±0.23	0.83±0.24	0.87±0.22	0.125
Deceleration time, ms	237.4±48.2	238.4±52.7	237.2±47.2	0.837
e’, cm/s	7.2±1.7	6.9±1.7	7.2±1.7	0.149
a’, cm/s	10.2±1.8	9.9±1.7	10.2±1.8	0.09
s’, cm/s	7.5±1.3	7.3±1.5	7.6±1.3	0.062
E/e’	9.1±2.9	9.6±3.0	9.0±2.8	0.068
**LV 3D speckle tracking echocardiography**				
3D-EF, %	53.8±6.0	52.4±7.1	54.0±5.7	**0.035**
3D-EF<50%, n[%]	90[17.0]	22[23.9]	68[15.6]	0.053
3D-EDV, ml/m^2^	57.3±13.3	61.3±17.0	56.5±12.2	**0.011**
3D-ESV, ml/m^2^	26.7±8.4	29.7±12.7	26.0±7.0	**0.008**
3D-SV, ml/m^2^	30.6±7.0	31.6±7.3	30.4±7.0	0.168
3D-GLS, %	-19.0±3.0	-18.2±3.3	-19.2±2.9	**0.008**
3D-GCS, %	-25.6±4.1	-24.9±4.6	-25.8±4.0	0.096
Averaged peak 3D-LS, %	-18.3±2.9	-17.5±3.1	-18.5±2.9	**0.004**
Averaged peak 3D-CS, %	-25.8±4.1	-25.0±4.8	-25.9±4.0	0.089
Averaged peak 3D-RS, %	37.0±5.2	35.6±6.1	37.3±4.9	**0.015**
Averaged peak 3D-PTS, %	-31.1±4.1	-30.2±4.8	-31.3±3.9	**0.026**
Global twist, °	13.4±6.9	13.4±6.9	13.1±7.3	0.784
Global torsion, °/cm	1.65±0.88	1.67±0.88	1.57±0.84	0.385

Data are mean±SD, median (interquartile range) or n(%).

Abbreviations: BSA, body surface area; CS, circumferential strain; EDV, end-diastolic volume; EF, ejection fraction; ESV, end-systolic volume; HDL, high-density lipoprotein; GCS, global circumferential strain; GLS, global longitudinal strain; IVSd, diastolic interventricular septum thickness; LS, longitudinal strain; LV, left ventricular; LVIDd, diastolic LV internal diameter; LVIDs, systolic LV internal diameter; MACE, major adverse cardiac endpoints; PTS, principle tangential strain; PWd, diastolic posterior wall thickness; RS, radial strain; and SV, stroke volume.

### Relations of myocardial deformation with long-term outcomes

**Fig 3:**
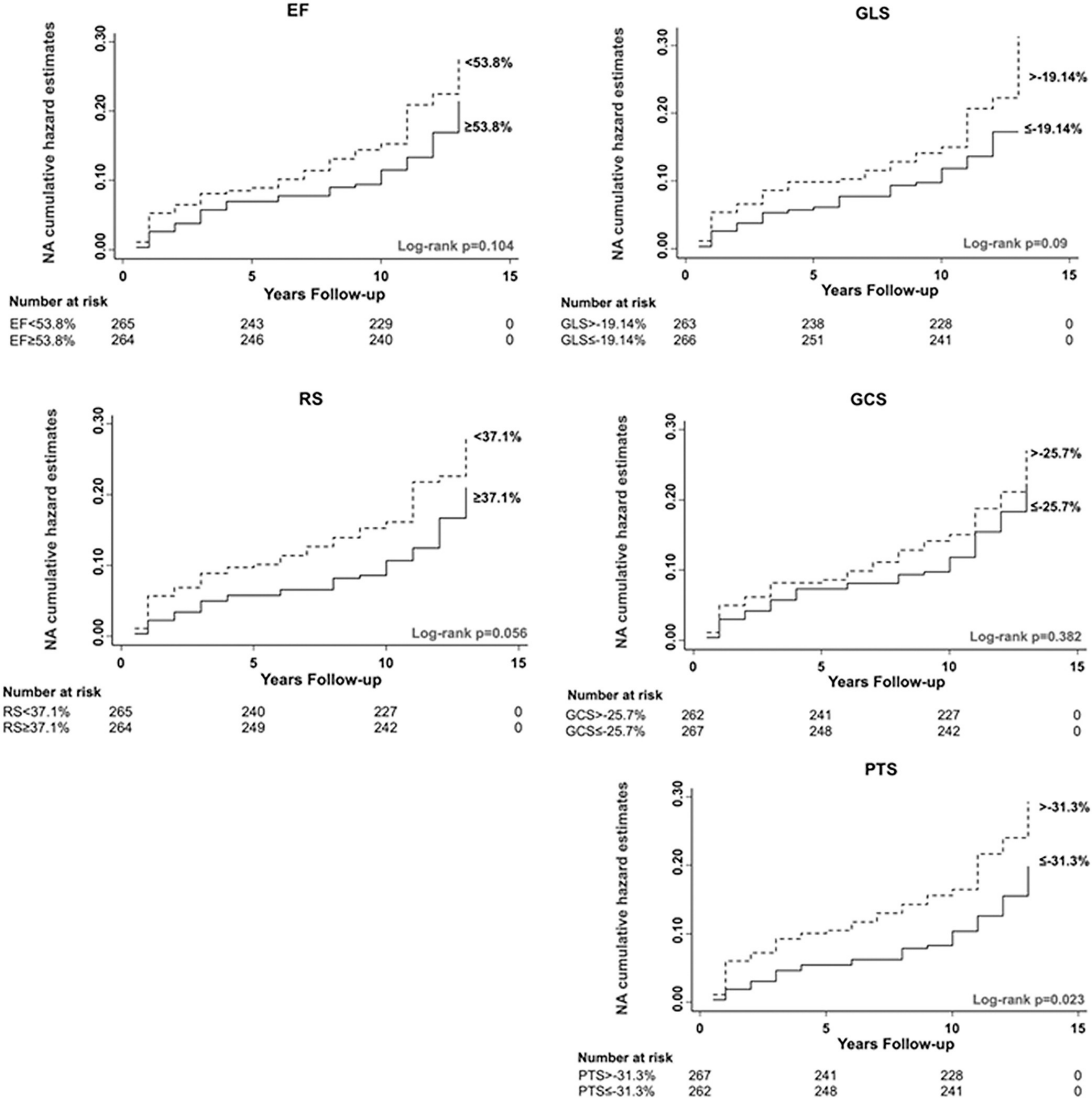
Unadjusted survival analysis of predictors. Nelson-Aalen cumulative hazard curves by medians of 3D-STE LV indices (major adverse cardiac endpoint). Dashed line = ≥median, solid line = <median. Abbreviations: EF, ejection fraction; GCS, global circumferential strain; GLS, global longitudinal strain; PTS, principle tangential strain; and RS, radial strain.

Nelson-Aalen cumulative hazard curves for 3D-STE LV myocardial strains and 3D-EF vs. MACE are shown in **Fig 3.** The prevalence of MACE for each stratum is summarized in **S2 Table in S1 File**. Nelson-Aalen cumulative hazard curves for global twist and torsion are shown in (**S1** and **S2 Figs in S1 File**).

In unadjusted Cox analyses, 3D-EF, 3D-GLS, 3D-PTS and 3D-RS were associated with MACE (**Table 2** and **Fig 3**). Evidence of an association between 3D-GCS and MACE was doubtful and evidence for 3D twist and torsion was unconvincing. Associations for 3D-GLS, 3D-PTS, 3D-RS and 3D-EF were little altered following adjustment for CVDRF and LV-3DE image-quality score (model-2). Further adjustment for prior history of CHD slightly attenuated the associations (model-3: **Table 2**). After inclusion of 2D-EF in models (model-3: **Table 2**) all associations between 3D-STE LV functional indices were attenuated to the null.

**Table 2:** Associations between major adverse cardiovascular endpoints ((n = 92) and 3D-STE LV functional indices in the overall population.

	Unadjusted (n = 529)	Model-1 (n = 529)	Model-2 (n = 523)	Model-3 (n = 523)	Model-4 (n = 517)
	Standardized HR (95% CI), p value	Standardized HR (95% CI), p value	Standardized HR (95% CI), p value	Standardized HR (95% CI), p value	Standardized HR (95% CI), p value
**3D-EF, %**	0.77(0.64,0.94),0.009	0.82(0.67,1.01),0.062	0.80(0.65,1.0),0.041	0.85(0.69,1.05),0.131	1.02(0.77,1.35),0.887
*C-statistic (95% CI)*	*0*.*57(0*.*51*,*0*.*64)*				
**3D-GLS, %**	0.74(0.60,0.90),0.003	0.81(0.66,1.00),0.046	0.79(0.65,0.98),0.029	0.83(0.68,1.02),0.086	0.96(0.75,1.23),0.756
*C-statistic (95% CI)*	*0*.*58(0*.*52*,*0*.*65)*				
**3D-GCS, %**	0.83(0.67,1.02),0.069	0.88(0.71,1.10),0.286	0.86(0.69,1.09),0.218	0.90(0.73,1.14),0.414	1.09(0.82,1.45),0.554
*C-statistic (95% CI)*	*0*.*55(0*.*49*,*0*.*62)*				
**3D-PTS, %**	0.75(0.62,0.90),0.006	0.82(0.67,1.01),0.064	0.80(0.65,1.0),0.038	0.85(0.69,1.04),0.117	0.96(0.73,1.25),0.781
*C-statistic (95% CI)*	*0*.*58(0*.*51*,*0*.*65)*				
**3D-RS, %**	0.74(0.61,0.90),0.003	0.80(0.65,0.98),0.036	0.77(0.63,0.96),0.019	0.83(0.67,1.03),0.086	1.00(0.76,1.31),0.999
*C-statistic (95% CI)*	*0*.*58(0*.*51*,*0*.*65)*				
**3D Twist, °**	0.97(0.76,1.23),0.80	0.99(0.77,1.27),0.94	0.97(0.75,1.25),0.79	0.98(0.77,1.26),0.90	1.04(0.80,1.34),0.775
*C-statistic (95% CI)*	*0*.*52 (0*.*44*,*0*.*59)*				
**3D Torsion, °/cm**	0.90(0.71,1.16),0.423	0.92(0.71,1.20),0.557	0.91(0.70,1.19),0.502	0.93(0.72,1.21),0.589	1.00(0.76,1.31),0.983
*C-statistic (95% CI)*	*0*.*53(0*.*46*,*0*.*60)*				

Abbreviations are as in Table 1 in addition to CI, confidence interval. Model 1: Adjusted for age, sex and ethnicity. Model 2: Model-1+hypertension, body mass index, diabetes mellitus, cystatin-C, smoking and LV-3DE image-quality score. Model 3: Model-2+prior history of coronary heart disease. Model 4: Model 2+2D ejection fraction. 3D-*EF*, *SD = 6*.*0%; 3D-GLS*, *SD = 3*.*0%; 3D-GCS*, *SD = 4*.*1%;3D- PTS*, *SD = 4*.*1%;3D- RS*, *SD = 5*.*2% Twist SD = 6*.*9°*, *Torsion SD = 0*.*88°/cm*. Note that HR for 3D-GLS, 3D-CLS and 3D-PTS were calculated after multiplying strain values by -1 to facilitate comparison of HR for all predictors.

In unadjusted models, 3D-PTS, 3D-GLS and 3D-RS were very slightly stronger discriminators of MACE than 3D-EF (**Table 2**); however, differences in C-statistic between all 3D-STE derived strain indices and 3D-EF were negligible.

Results were similar in models additionally adjusted for HtR (**S3 Table in S1 File**) and accounting for non-cardiovascular deaths as a competing risk had little effect on estimates (**S4 Table in S1 File**). There was no evidence that prior history of CHD modified the associations between the outcome, and 3D-STE derived indices. However, associations with the outcome were attenuated after excluding individuals with prior history of CHD, such that there was no convincing evidence of associations with MACE (**Table 3**), although due to the reduced number of events 95% CI were wide and clinically important associations could not be excluded.

**Table 3:** Associations between major adverse cardiac endpoints (n = 68) and 3D-STE LV functional indices in the overall population without prior history of CHD (n = 475).

	Unadjusted	Model-1	Model-2 (n = 469)
	Standardized HR (95% CI), p value	Standardized HR (95% CI), p value	Standardized HR (95% CI), p value
**3D-EF, %**	0.86(0.67, 1.10), 0.236	0.91(0.69, 1.19), 0.504	0.88(0.66, 1.17), 0.374
**3D-GLS, %**	0.79(0.61, 1.01), 0.057	0.84(0.65, 1.09), 0.192	0.83(0.64, 1.09), 0.170
**3D-GCS, %**	0.94(0.73, 1.20), 0.641	1.01(0.78, 1.31), 0.924	0.99(0.74, 1.31), 0.926
**3D-PTS, %**	0.86(0.67, 1.1), 0.262	0.93(0.71, 1.22), 0.611	0.90(0.68, 1.20), 0.498
**3D-RS, %**	0.83(0.64, 1.06), 0.140	0.88(0.67, 1.15), 0.356	0.84(0.63, 1.13), 0.246
**3D Twist, °**	1.17(0.88, 1.55), 0.270	1.20(0.89, 1.61), 0.237	1.18(0.86, 1.60), 0.308
**3D Torsion, °/cm**	1.06(0.80, 1.42), 0.654	1.10(0.81, 1.49), 0.553	1.09(0.80, 1.51), 0.572

Abbreviations and footnotes are as in Table 2. *3D-EF*, *SD = 5*.*8%; 3D-GLS*, *SD = 2*.*9%; 3D-GCS*, *SD = 4*.*0%; 3D-PTS*, *SD = 3*.*9%; 3D-RS*, *SD = 4*.*9%*, *Twist SD = 6*.*8°*, *Torsion SD = 0*.*87°/cm*. Note that HR for 3D-GLS, 3D-CLS and 3D-PTS were calculated after multiplying strain values by -1 to facilitate comparison of HR for all predictors.

### Incremental value of 3D-EF, 3D-GLS and 3D-PTS in relation to predicting future outcomes in the general population

Inclusion 3D-EF, 3D-GLS and 3D-PTS increased the goodness of fit of the model when compared with FRS, but the improvement was modest (**Table 4**). Inclusion of both 3D-GLS and 3D-PTS slightly improved the discrimination and calibration compared with 3D-EF (**Table 4**) but differences between all models, including the FRS model alone, were small. There was no convincing evidence that adding any of these measures improved continuous NRI or IDI (**Table 4**), although the 95% CI were wide, and it was not possible to exclude clinically important effects.

**Table 4:** The incremental prognostic value of 3D-EF, 3D-GLS, and 3D-PTS over FRS.

	Clinical model	Clinical model + 3D-EF	Clinical model + 3D-GLS	Clinical model + 3D-PTS
**Goodness-of-fit/model calibration**				
**Overall population (n = 523)**				
**Major adverse cardiac endpoint (n = 92)**				
Chi-square (change vs. baseline)	37.0 (baseline)	+3.8	+5.2	+4.6
P-value vs. baseline	-	**0.047**	**0.022**	**0.032**
AIC/BIC	1098.8/1137.1	1097.0/1139.6	1095.5/1138.1	1096.1/1138.7
*C-statistic(95% CI)*	0.698(0.647, 0.749)	0.709(0.656, 0.763)	0.715(0.663, 0.766)	0.712(0.659, 0.766)
*Continuous NRI(95% CI)*	-	0.109(-0.261, 0.347)	0.128(-0.200, 0.381)	0.157(-0.221, 0.387)
*IDI(95% CI)*	-	0.004(-0.004, 0.031)	0.005(-0.003, 0.036)	0.007(-0.004, 0.040)
**Population after excluding individuals with prior history of CHD (n = 469)**				
**Major adverse cardiac endpoint (n = 68)**				
Chi-square (change vs. baseline)	30.0 (baseline)	+0.42	+2.1	+0.43
P-value vs. baseline	-	0.517	0.148	0.512
AIC/BIC	799.1/836.4	800.6/842.1	798.98/840.486	800.6/842.1
*C-statistic(95% CI)*	0.689(0.638, 0.740)	0.698(0.648, 0.748)	0.705(0.655, 0.755)	0.701(0.651, 0.751)
*Continuous NRI(95% CI)*	-	-0.045(-0.328, 0.349)	0.062(-0.310, 0.358)	-0.208(-0.347, 0.331)
*IDI(95% CI)*	-	-0.003(-0.006, 0.018)	0.001(-0.005, 0.026)	-0.002(-0.005, 0.018)

Clinical model (Framingham risk score): Age, sex, anti-hypertensive medications, diabetes mellitus, systolic blood pressure, body mass index, cholesterol, smoking. BIC, Bayesian information criterion; AIC, Akaike’s information criterion; NRI, net reclassification improvement; IDI, integrated discrimination improvement.

In a sensitivity analysis, there was no evidence of incremental predictive value of 3D-EF, 3D-GLS and 3D-PTS over FRS for the prediction of MACE after exclusion of individuals with prior history of CHD (**Table 4**).

## Discussion

3D-STE is increasingly used to assess LV function; however, its low temporal and spatial resolution, its strong dependence on image quality and frame rates [20,21], and inter-vendor differences in analysis methods are well-recognised limitations [8,10,11,22]. Existing evidence on the utility of 3D-STE is largely limited to diseased populations and its role in the community or general population is uncertain. Previously we have reported that 3D-STE had poor feasibility in the setting of a multi-ethnic community sample [13,14]. We now report the long-term prognostic significance of 3D-STE derived LV myocardial strain indices in the same study. We demonstrate the following: (1) 3D-GLS, 3D-PTS and 3D-RS were more impaired in participants with MACE compared to those without. (2) 3D-GLS, 3D-PTS, 3D-RS predicted MACE and HR and C-statistics were marginally larger than for 3D-EF; however associations of MACE with 3D-EF 3D-GLS, 3D-PTS, 3D-RS were almost completely abolished in models adjusted for 2D-EF, (3) 3D-PTS and 3D-GLS added incremental prognostic value to FRS, with equivocal evidence that they were slightly superior to 3D-EF, but with no convincing evidence of improved reclassification or discrimination.

Previous studies, mostly using 2D-STE, have proposed that GLS may be a more sensitive measure of LV systolic dysfunction than EF, particularly in patients with mild disease [5]. Some recent guidelines advocate using both EF and GLS by 2D-STE for LV function quantification [6,7], but there is little evidence regarding 3DE. Medvedofsky et al., found that GLS by 3D-STE might be a more robust prognosticator when compared to more common echocardiographic indices (i.e. 2D-EF and GLS by 2DE) [9]. Our data suggests that any prognostic advantage of 3D-STE GLS is likely to be small in the general population who are at lower risk of events.

3D-RS was associated with increased incidence of MACE whereas associations with 3D-CS were unconvincing. Using 2D-STE Cheng, et al. found no evidence of a relation between RS or GCS with CHD [23]. In addition to differences in the strain analysis methods employed, [24] differences might be explained by the fact that the Framingham Offspring Study (FOS) restricted its sample to individuals without any prevalent CVD at baseline, whereas our population-based sample included people who had a prior history of CHD. Consistent with this, participants in SABRE had poorer LV systolic function on average than participants in FOS and excluding people with prior CHD weakened associations in SABRE.

3D-PTS (also called 3D strain) is a novel measure which reflects contributions from multiple directional deformations simultaneously, potentially decreasing tracking errors because of higher signal to noise ratio and providing a better assessment of the overall magnitude of deformation [16,25]. A few studies have examined the prognostic value of 3D-STE derived strain in diseased populations. Shine et al. found that 3D-PTS was independently associated with increased risk of deaths or HF in 96 patients after acute MI studied over a median follow-up of 33±10 months [10]. In a high-risk sample of 163 patients undergoing cardiac surgery, Howard-Quijano et al. found that preoperative 3D-PTS was an independent predictor of acute and long-term clinical outcomes after cardiac surgery [22]. In keeping with these findings, we found that 3D-PTS was predictive of MACE independent of CVDRF, but adjustment for 2D-EF abolished associations between 3D-PTS and MACE. 3D-PTS did not convincingly demonstrate improved reclassification/discrimination.

### Study strengths and limitations

A strength of the present study is its prospective, community-based design and the inclusion of a multi-ethnic sample of older men and women with long-term median follow-up of more than a decade. A limitation of this study is the relatively small number of events and the consequent limited statistical power. The use of a composite endpoint may obscure associations for infrequent but important outcomes e.g. HF. The large proportion of people who did not have acceptable 3D-STE is another limitation. Not counting people who attended clinic before the 3D probe was available, acceptable 3D-STE was achieved in only 38% of participants. 47% of participants who had acceptable 3DE did not have acceptable 3D-STE because of a variety of problems, including inadequate frame rate, major stitch artifact, poor ECG signals or inadequate image quality [13]. 3D-STE was more frequently not feasible in older participants, South Asian people, or individuals with higher BMI, hypertension, DM, or a history of CHD. This will introduce bias, but reflects the feasibility of 3D-STE, at least between 2008 and 2011 when the study was performed. While the technology was state-of-the-art at the time, 3D-STE methodology has improved recently so our study may underestimate feasibility. 3D-STE is also sensitive to the quality of acceptable images [14], but in this study, image quality was evaluated thoroughly, and adjustment for image quality had little effect on associations.

## Conclusions

In a multi-ethnic sample of older people, selected 3D-STE indices of LV myocardial mechanics predicted MACE independently of clinical cardiovascular risk factors but not 2D-EF. 3D-GLS and 3D-PTS were marginally superior to 3D-EF in predicting MACE, but with no convincing evidence of improved reclassification/discrimination. LV myocardial strains measured by 3D-STE adds little prognostic information in elderly community-dwelling individuals.

## Supporting information

S1 File S1 –S4 Tables and S1 & S2 Figs. (DOCX)
