# Supplementary material for: Does 3D-speckle tracking echocardiography improve prediction of major cardiovascular events in a multi-ethnic general population? A Southall and Brent Revisited (SABRE) cohort study
Source: PLoS One. 2023 Jun 27;18(6):e0287173. doi: 10.1371/journal.pone.0287173 (PMC10298788; doi:10.1371/journal.pone.0287173)
Supplement: S1 File — (DOCX) [file pone.0287173.s001.docx]

**Supporting information**

**Table-S1: Baseline characteristics of SABRE participants with and without 3D-STE.**

**Table-S2: The prevalence of major adverse cardiac endpoints by the median of 3D-STE-derived LV functional indices.**

**Table-S3: Sensitivity analysis: Associations of major adverse cardiac endpoints (n=92) with 3D-STE-derived LV functional indices in the overall population (n=529) after adjustment for heart rate.**

**Table-S4: Associations between major adverse cardiac endpoints (n=92) and 3D-STE-derived LV functional indices in the overall population (n=529; competing risk analysis.**

**Figure S1: Nelson-Aalen cumulative hazard curves by medians of twist.**

**Figure S2: Nelson-Aalen cumulative hazard curves by medians of torsion.**

| **Table-S1 Baseline characteristics of SABRE participants with and without 3D-STE** | | | |
| --- | --- | --- | --- |
|  | **+ 3D-STE (n=529)** | **- 3D-STE (n=878)** | ***P*** |
| **Clinical variables** |  |  |  |
| **Demographics** |  |  |  |
| Age, y | 69.1±6.1 | 70.0±6.1 | **0.009** |
| Male, n(%) | 405(76.6) | 664(75.6) | 0.69 |
| Ethnicity, n(%) |  |  | **<0.0001** |
| Europeans | 273(51.6) | 396(45.1) |  |
| South Asians | 151(28.5) | 359(40.9) |  |
| African Caribbean | 105(20.0) | 123(14.0) |  |
| **Clinical history** |  |  |  |
| Systolic blood pressure, mmHg | 140.2±17.9 | 140.1±17.8 | 0.96 |
| Diastolic blood pressure, mmHg | 76.5±9.6 | 77.3±9.8 | 0.14 |
| Heart rate, bpm | 67.2±11.4 | 68.9±12.7 | **0.008** |
| Body mass index, kg/m2 | 26.1±3.5 | 28.5±5.2 | **<0.0001** |
| Waist: hip ratio | 0.96±0.1 | 0.99±0.1 | **<0.0001** |
| Hypertension, n(%) | 301(56.9) | 642(73.1) | **<0.0001** |
| Known diabetes, n(%) | 118(22.3) | 322(36.7) | **<0.0001** |
| Prior coronary heart diseases, n(%) | 54(10.2) | 247(28.1) | **<0.0001** |
| Smoking status, n(%) never/ex/current |  |  | 0.09 |
| Never | 285(54.1) | 511(58.7) |  |
| Ex. | 201(38.1) | 314(36.0) |  |
| Current | 41(7.8) | 46(5.3) |  |
| **Medications** |  |  |  |
| Anti-diabetic drugs, n(%) | 71(13.4) | 231(26.3) | **<0.0001** |
| Lipid lowering drugs, n(%) | 247(46.7) | 537(61.2) | **<0.0001** |
| **Laboratory work** |  |  |  |
| Fasting blood triglycerides, mmol/l | 1.0(0.8-1.4) | 1.2(0.9-1.6) | **<0.0001** |
| Fasting blood cholesterol: HDL ratio | 3.6±1.0 | 3.6±1.0 | 0.44 |
| Cystatin C, mg/l | 1.0±0.3 | 1.1±0.3 | **<0.0001** |
| HbA1c, % | 6.1±0.9 | 6.4±1.1 | **<0.0001** |
| ProBNP, pg/ml | 80[46-144] | 101[52-221] | **<0.0001** |
| Troponin, pg/ml | 6.5[4.0-9.9] | 7.7[5.0-12.4] | **<0.0001** |
| **Conventional echocardiography** | |  |  |
| LVIDd, cm | 4.4±0.42 | 4.5±0.49 | **0.010** |
| LVIDs, cm | 2.9±0.45 | 3.0±0.53 | **0.0004** |
| IVSd, cm | 1.1±0.20 | 1.2±0.21 | **<0.0001** |
| PWd, cm | 1.0±0.16 | 1.0±0.17 | **0.0004** |
| Relative wall thickness | 0.46±0.08 | 0.47±0.09 | **0.024** |
| EF, % | 62.5±9.4 | 60.9±10.3 | **0.003** |
| EDV, ml/m^2^ | 49.6±9.9 | 50.3±11.6 | 0.25 |
| ESV, ml/m^2^ | 18.8±7.3 | 20.0±8.5 | **0.005** |
| LV mass indexed to BSA, g/m2 | 92.3±22.4 | 97.4±23.4 | **0.0001** |
| LV mass indexed to height^2.7^, g/h^2.7^ | 41.5±10.8 | 46.0±12.8 | **<0.0001** |
| E wave, cm/s | 62.7±15.6 | 64.3±18.3 | 0.09 |
| A wave, cm/s | 74.8±16.4 | 76.9±19.2 | **0.033** |
| E/A ratio | 0.86±0.23 | 0.85±0.25 | 0.32 |
| Deceleration time, ms | 237.4±48.1 | 241.1±53.3 | 0.18 |
| Average e’, cm/s | 7.1±1.7 | 7.1±1.9 | 0.32 |
| Average a’, cm/s | 10.1±1.8 | 10.1±2.0 | 0.53 |
| Average s’, cm/s | 7.5±1.3 | 7.4±1.5 | 0.30 |
| E/e’ | 9.1±2.9 | 9.7±3.6 | **0.004** |
| Data are mean±SD, median (interquartile range) or n(%). Abbreviations: BSA, body surface area; EDV, end-diastolic volume; EF, ejection fraction; ESV, end-systolic volume; HDL, high-density lipoprotein; IVSd, diastolic interventricular septum thickness; LV, left ventricular; LVIDd, diastolic LV internal diameter; LVIDs, systolic LV internal diameter; and PWd, diastolic posterior wall thickness. | | | |

| **Table-S2 The prevalence of major adverse cardiac endpoints by the median of 3D-STE-derived LV functional indices** | | |
| --- | --- | --- |
|  | **≥median** | **<median** |
| **3D-EF, median=53.8%** | 39(14.8%) | 53(20.0%) |
| **3D-GLS, median=-19.14%** | 39(14.7%) | 53(20.2%) |
| **3D-GCS, median=-25.7%** | 43(16.1%) | 49(18.7%) |
| **3D-PTS, median=-31.3%** | 36(13.7%) | 56(21.0%) |
| **3D-RS, median=37.1%** | 38(14.4%) | 54(20.4%) |
|  |  |  |
|  |  |  |
| Abbreviations: EF, ejection fraction; GCS, global circumferential strain; GLS, global longitudinal strain; PTS, principle tangential strain; and RS, radial strain. | | |
|  | | |

| **Table-S3 Sensitivity analysis: Associations of major adverse cardiac endpoints (n=92) with 3D-STE-derived LV functional indices in the overall population (n=529) after adjustment for heart rate.** | |
| --- | --- |
|  | **Standardized HR (95% CI), p value** |
| **3D-EF, %** | 0.83(0.67, 1.03), 0.086 |
| **3D-GLS, %** | 1.22(1.0, 1.50), 0.058 |
| **3D-GCS, %** | 1.12(0.89, 1.40), 0.333 |
| **3D-PTS, %** | 1.20(0.97, 1.47), 0.095 |
| **3D-RS, %** | 0.81(0.66, 1.01), 0.058 |
|  |  |
|  |  |
| Abbreviations as in table-S2. Adjusted for age, sex, ethnicity, hypertension, body mass index, diabetes mellitus, Cystatin-C, smoking, LV-3DE image-quality score, history of coronary heart disease and heart rate. *3D-EF, SD=6.0%; 3D-GLS, SD=3.0%; 3D-GCS, SD=4.1%; 3D-PTS, SD=4.1%; 3D-RS, SD=5.2%.* | |

| Table-S4 Associations between major adverse cardiac endpoints (n=92) and 3D-STE-derived LV functional indices in the overall population (n=529; competing risk analysis) | | | | | |
| --- | --- | --- | --- | --- | --- |
|  | **Unadjusted** | | **Model-1** | **Model-2 (n=523)** | **Model-3 (n=523)** |
|  | **Standardized HR (95% CI), p value** | | **Standardized HR (95% CI), p value** | **Standardized HR (95% CI), p value** | **Standardized HR (95% CI), p value** |
| **3D-EF, %** | | 0.77(0.63, 0.95), 0.015 | 0.83(0.66, 1.04), 0.098 | 0.80(0.63, 1.02), 0.075 | 0.85(0.68, 1.07), 0.178 |
| **3D-GLS, %** | | 1.35(1.10, 1.66), 0.004 | 1.23(0.99, 1.52), 0.058 | 1.26(1.0, 1.56), 0.041 | 1.19(0.97, 1.47), 0.102 |
| **3D-GCS, %** | | 1.21(0.96, 1.52), 0.102 | 1.12(0.88, 1.43), 0.358 | 1.15(0.88, 1.50), 0.295 | 1.09(0.85, 1.40), 0.474 |
| **3D-PTS, %** | | 1.33(1.06, 1.65), 0.012 | 1.22(0.96, 1.54), 0.104 | 1.25(0.99, 1.58), 0.064 | 1.18(0.95, 1.46), 0.144 |
| **3D-RS, %** | | 0.74(0.60, 0.92), 0.006 | 0.80(0.64, 1.01), 0.062 | 0.77(0.61, 0.98), 0.037 | 0.83(0.66, 1.05), 0.118 |
|  | |  |  |  |  |
|  | |  |  |  |  |
| n=29 competing events (non-CV deaths); n=28 for model-2 and model-3. | | | | | |
| Abbreviations as in table-S2. Model 1: adjusted for age, sex and ethnicity. Model 2: model-1+hypertension, body mass index, diabetes mellitus, Cystatin-C, smoking and image quality. Model 3: model-2+history of coronary heart disease. *3D-EF, SD=6.0%; 3D-GLS, SD=3.0%; 3D-GCS, SD=4.1%; 3D-PTS, SD=4.1%; 3D-RS, SD=5.2%.* | | | | | |

|  |
| --- |
| Figure S1: Nelson-Aalen cumulative hazard curves by medians of twist. |

|  |
| --- |
| Figure S2: Nelson-Aalen cumulative hazard curves by medians of torsion. |
